# Perceived Social Support and Quality of Life in Older Adults After Percutaneous Coronary Intervention: The Mediating Roles of Coping and Health Literacy

**DOI:** 10.3390/nursrep16070225

**Published:** 2026-06-29

**Authors:** Mengjing Sun, Fengjuan Shi, Juxia Wang, Zengfeng Su, Xiaojun Feng, Huiqin Sun

**Affiliations:** 1 School of Nursing, Anhui Medical University, Hefei 230032, China; 2345011308@stu.ahmu.edu.cn (M.S.);; 2 Department of Nursing, Chaohu Hospital Affiliated, Anhui Medical University, Hefei 238000, China; 3 Department of Rehabilitation Medicine, The Second Affiliated Hospital, Anhui Medical University, Hefei 230032, China

**Keywords:** aged, coronary heart disease, percutaneous coronary intervention, perceived social support, mediating effect

## Abstract

**Background**: Older adults undergoing percutaneous coronary intervention (PCI) often experience substantial physical and psychosocial challenges that may adversely affect their quality of life. Perceived social support, coping, and health literacy are important factors associated with health outcomes, yet the pathways linking these variables remain insufficiently understood. This study aimed to investigate the relationship between perceived social support and quality of life in older adults after PCI and to examine the mediating roles of coping and health literacy. **Methods**: This cross-sectional study included 353 older adults with coronary heart disease who underwent their first PCI at the cardiology department of a tertiary general hospital in Anhui Province, China. Data were collected using a general information questionnaire, the Perceived Social Support Scale, the Medical Coping Modes Questionnaire, the Health Literacy Scale for Patients Undergoing Percutaneous Coronary Intervention, and the Chinese Questionnaire for Quality of Life in Patients with Cardiovascular Disease. Pearson correlation analysis, hierarchical regression analysis, and structural equation modeling were used for data analysis. **Results**: The mean quality of life score was 82.20 ± 19.19. Perceived social support, coping, and health literacy were all positively associated with quality of life (all *p* < 0.01). Perceived social support had a direct positive effect on quality of life (effect = 0.071, [95% CI (0.012, 0.207)]). It also had indirect effects through coping (effect = 0.109, [95% CI (0.052, 0.174)]) and health literacy (effect = 0.511, [95% CI (0.191, 0.873)]). In addition, coping and health literacy showed a significant serial mediating effect in the association between perceived social support and quality of life (effect = −0.065, [95% CI (−0.236, −0.015)]). **Conclusions**: Perceived social support was associated with better quality of life in older adults after PCI, both directly and indirectly through coping and health literacy. These findings suggest that nursing care should pay greater attention to strengthening social support, encouraging positive coping, and improving health literacy in order to promote postoperative recovery and quality of life.

## 1. Introduction

Coronary heart disease (CHD) is the cardiac condition characterized by myocardial ischemia and hypoxia resulting from lumen narrowing, spasm, or obstruction caused by coronary atherosclerosis. With the acceleration of societal aging, individuals aged 65 and older now constitute 13.50% of China’s total population [1], and CHD remains highly prevalent among older adults and high-risk cardiovascular populations [2]. Percutaneous coronary intervention (PCI) serves as a key approach for managing coronary heart disease. Although it offers advantages such as minimal trauma and rapid recovery [3], patients face a sudden death risk 4 to 6 times higher than the general population post-procedure [4]. Furthermore, quality of life serves as a crucial indicator for assessing patient health status and plays a significant predictive role in treatment outcomes and disease prognosis [5]. Additionally, quality of life is closely associated with patients’ ability to perceive social support, coping strategies for illness, and health literacy [6,7]. Perceived social support includes the emotional feelings and satisfaction of being valued, supported, and understood by other community members. It serves as a key factor in promoting psychosocial adaptation and is a crucial indicator of patients’ cognitive abilities. However, CHD patients face a high risk of cognitive impairment, reaching up to 45%. Previous studies have shown that patients after PCI generally perceive a moderate level of social support, and higher perceived social support is associated with better quality of life, suggesting that social support may be an important psychosocial factor influencing quality of life after PCI [8,9]. Cognitive dysfunction can weaken postoperative older adults’ comprehension and perception abilities [10]. Resilience against neurodegenerative diseases and psychosocial motivators are crucial for maintaining brain cognitive function, enabling patients to actively engage in post-surgical cardiac rehabilitation. Medical coping strategies are characterized by cognitive adjustment and environmental adaptation [11]. Confrontation coping, a problem-oriented coping strategy, may enhance disease management initiative in older adults after PCI and facilitate the acquisition and utilization of health information and resources, thereby improving health literacy [12,13]. Health literacy is the capacity of a person to access, comprehend, and evaluate essential health information in order to make well-informed choices [14]. Higher levels of perceived social support may promote positive coping strategies, improve the ability to access and utilize health information, and thereby contribute to better health outcomes in patients [15,16]. Although previous studies have demonstrated associations among perceived social support, coping strategies, health literacy, and quality of life, the underlying mechanisms among these variables in older adults undergoing PCI remain unclear. Therefore, based on the Stress-Appraisal-Coping model, this study aimed to examine the relationships among perceived social support, the confrontation dimension of medical coping, health literacy, and quality of life, and to explore the mediating roles of coping and health literacy. The findings may provide evidence for optimizing postoperative health management in older adults with coronary heart disease.

## 2. Objects and Methods

### 2.1. Research Subjects

Older adults with coronary heart disease who were undergoing their initial PCI procedure in the Cardiovascular Department of a tertiary general hospital located in Anhui Province were selected through convenience sampling from September 2024 to April 2025. Inclusion criteria: (1) Age ≥ 65 years old; (2) First-time PCI procedure; (3) Informed consent and voluntary participation. Exclusion criteria: (1) New York Heart Association functional level IV; (2) Concurrent major organ diseases (e.g., cardiac, cerebral); (3) Severe cognitive impairment or inability to communicate effectively. According to the requirements for analyzing statistical variables, the sample size for a cross-sectional survey needs to be a minimum of 5 to 10 times greater than the number of variables involved. The 15 variables were included in this study. Taking into account an 80% questionnaire validity rate requirement and based on a calculation that uses 10 times the number of variables, the minimum necessary sample size was determined to be 188 cases. Ultimately, 353 valid questionnaires were collected, meeting the structural equation modeling requirement of no fewer than 200 cases. Ethical approval for this study was granted by the hospital ethics committee (Approval No.: KYXM-202407-010).

### 2.2. Method

#### 2.2.1. Survey Tool

The basic information of patients was collected using a self-designed general information questionnaire. The main contents included sociodemographic characteristics and disease-related information. Sociodemographic data included age, gender, height, weight, dietary habits, etc. Disease-related information included the timing of surgery (emergency or elective PCI), left ventricular ejection fraction, low-density lipoprotein cholesterol level, cardiac function classification, fall risk, and the presence of comorbid chronic diseases. The severity of coronary artery lesions was assessed using the Gensini scoring system based on coronary angiography findings obtained from the electronic medical records [17].

The Perceived Social Support Scale (PSSS), developed by Zimet et al. [18], was used in its Chinese version revised by Qianjin Jiang [19], which has demonstrated good reliability and validity in Chinese populations. The measurement tool includes three components: family assistance, support from friends, and additional support, culminating in a total of 12 items. Each item is evaluated using a 7-point Likert scale that ranges from “strongly disagree” to “strongly agree” with scores assigned from 1 to 7. The total score of the scale ranges from 12 to 84. Initially, Cronbach’s α coefficient was 0.800; however, in this research, it increased to 0.838.

The Medical Coping Modes Questionnaire, developed by Feifei and sinicized by Xiaohong Shen [20,21], was used to assess patients’ attitudes toward coping with illness. The questionnaire consists of three dimensions: confrontation, avoidance, and acceptance–resignation, with a total of 20 items. Each item is rated on a 4-point Likert scale ranging from 1 to 4. Higher scores on a given dimension indicate a greater tendency toward that coping style. Furthermore, the Cronbach’s α values for the dimensions of confrontation, avoidance, and acceptance-resignation were recorded as 0.799, 0.732, and 0.722, respectively. In this study, the Cronbach’s α coefficient for the confrontation dimension was measured at 0.718.

The Health Literacy Scale for Patients Undergoing Percutaneous Coronary Intervention (PCI), developed by Meng Yue [22], was used to assess the health literacy level of patients undergoing PCI. The scale consists of three dimensions: functional health literacy, communicative health literacy, and critical health literacy, comprising a total of 27 items. Each item is rated on a 5-point Likert scale ranging from 1 to 5, with four items reverse-scored. The total score ranges from 27 to 135, with higher scores indicating a higher level of health literacy. Cronbach’s α coefficient of the original scale was 0.824, and in the present study, the Cronbach’s α coefficient was 0.970.

The Chinese Questionnaire for Quality of Life in Patients with Cardiovascular Disease, developed by Jiangsheng Liu [23]. The tool comprises 24 questions organized into six categories: physical condition, health status, medical condition, overall life, psychological well-being, and capacity for work. The maximum achievable score is 154, where higher scores reflect an improved quality of life. Furthermore, this original questionnaire had a Cronbach’s α coefficient of 0.910; however, in the current study, it was recorded at 0.850, reflecting a satisfactory level of internal consistency.

#### 2.2.2. Survey Methods

A data collection team was established, consisting of one head nurse from the Department of Cardiology, one nurse in charge, and two postgraduate students. After obtaining informed consent, the questionnaires were administered one day before discharge using a one-to-one approach. The patients themselves filled out the questionnaires. In addition, participants with intact cognitive function but with reading or writing difficulties were assisted by trained investigators during questionnaire completion. The time needed for each participant to complete the questionnaire was roughly 10 to 30 min. Questionnaires were distributed and collected in person. Two researchers independently carried out data entry and conducted cross-checks to guarantee the accuracy, completeness, and validity of the information.

### 2.3. Statistical Analysis

All statistical analyses were performed using SPSS (version 25.0; IBM Corp., Armonk, NY, USA), and structural equation modeling (SEM) was conducted using AMOS (version 26.0; IBM Corp., Armonk, NY, USA). Continuous variables with a normal distribution were expressed as mean ± standard deviation (SD), whereas categorical variables were presented as frequencies and percentages. Group differences were analyzed using independent-samples *t*-tests or one-way analysis of variance (ANOVA), as appropriate. Pearson correlation analysis was performed to examine the relationships among perceived social support, the confrontation dimension of medical coping, health literacy, and quality of life. SEM was used to test the hypothesized mediation model. The significance of indirect effects was assessed using the bootstrap method with 5000 resamples. A mediating effect was considered statistically significant when the 95% confidence interval (CI) did not include zero. All statistical tests were two-tailed, and a *p*-value < 0.05 was considered statistically significant.

## 3. Results

### 3.1. Patient Demographics and Quality of Life Levels

Common method bias was assessed using Harman’s single-factor test. Fourteen factors with eigenvalues > 1 were extracted, and the first unrotated factor explained 28.77% of the total variance, which was below the threshold of 40%, indicating no significant common method bias.

This research involved 353 older adults suffering from coronary artery disease who received PCI, with ages between 65 and 91 years and a mean age of 71.91 ± 6.15 years. The univariate analysis indicated that factors such as age, gender, education level, monthly household income, heart function classification, and the severity of coronary artery disease significantly influenced patients’ quality of life, showing statistically meaningful differences (*p* < 0.05), as demonstrated in Table 1.

### 3.2. Patients’ Perception of Social Support, Medical Coping Dimensions, Health Literacy, and Quality of Life Levels

The overall quality of life measurement for older adults diagnosed with coronary heart disease who received PCI was 82.20 ± 19.19 points. The overall score for perceived social support was 54.50 ± 7.27 points, while the total score for the confrontation dimension of the Medical Coping Modes Questionnaire was 18.40 ± 3.58 points. Furthermore, the health literacy score was 76.69 ± 19.12 points, as shown in Table 2.

### 3.3. Analysis of the Correlation Between Patients’ Perceived Social Support, Medical Coping Dimensions, Health Literacy, and Quality of Life

Pearson correlation analysis showed that quality of life was positively correlated with perceived social support (r = 0.401, *p* < 0.01), the confrontation dimension of medical coping (r = 0.298, *p* < 0.01), and health literacy (r = 0.277, *p* < 0.01). Perceived social support was positively correlated with confrontation coping (r = 0.597, *p* < 0.01) and health literacy (r = 0.399, *p* < 0.01). In addition, confrontation coping was positively correlated with health literacy (r = 0.521, *p* < 0.01), as shown in Table 3.

### 3.4. Multivariate Linear Hierarchical Regression Analysis of Variable Relationships in Chain-of-Agency Models

In the hierarchical regression analysis, factors influencing patients’ quality of life were included as control variables. In Step 1, perceived social support was entered as the independent variable and the confrontation dimension of medical coping as the dependent variable. In Step 2, perceived social support and the confrontation dimension were entered as independent variables, with health literacy as the dependent variable. In Step 3, perceived social support, the confrontation dimension, and health literacy were entered as independent variables, and quality of life was set as the dependent variable. The variance inflation factors (VIF < 5) met the required criteria, indicating that there was no multicollinearity among the variables in each model. The results are shown in Table 4.

### 3.5. Model Development and Refinement

**Figure 1:**
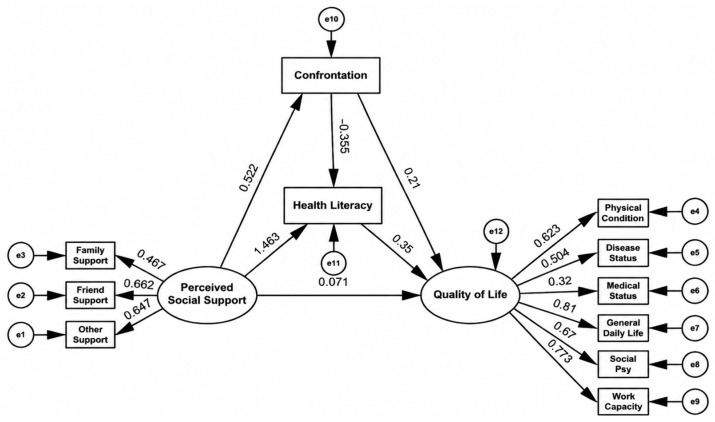
Chain Mediation Model of Medical Coping Dimensions and Health Literacy in Perceived Social Support and Quality of Life.

Based on the Stress and Coping Theory, a structural equation model was constructed with the confrontation dimension of medical coping and health literacy as sequential mediating variables, perceived social support as the independent variable, and quality of life as the dependent variable. Following the adjustments made to the model, the indices indicating goodness-of-fit were as follows: χ^2^/df = 3.127, NFI = 0.959 (>0.900), IFI = 0.972 (>0.900), TLI = 0.948 (>0.900), and RMSEA = 0.078 (<0.080). Each of these indices satisfied the suggested thresholds, signifying a strong fit for the model, as depicted in Figure 1.

### 3.6. Significance Test for Chain Mediation Effects

Bootstrapping analysis with 5000 resamples was performed to examine the significance of the mediation effects, and 95% confidence intervals (95% CIs) were calculated. A mediation effect was considered statistically significant when the 95% CI did not include zero. The results showed that the mediation effect of perceived social support on quality of life through the confrontation dimension of medical coping was 0.109 (95% CI: 0.052–0.174), accounting for 17.38% of the total effect, indicating a significant mediating role. The indirect effect through health literacy was 0.511 (95% CI: 0.191–0.873), accounting for 81.50% of the total effect, also indicating a significant mediating effect. The chain mediation pathway (perceived social support → confrontation coping → health literacy → quality of life) was −0.065 (95% CI: −0.236 to −0.015), indicating a significant serial mediation effect with a suppression effect. According to mediation effect theory, when the indirect effect has an opposite sign to the direct effect, a suppression effect is indicated. In this study, the direct effect was positive (β = 0.071), while the chain indirect effect was negative (β = −0.065), suggesting a suppression effect in this pathway. This finding indicates that confrontation coping and health literacy may partially suppress the positive association between perceived social support and quality of life, as shown in Table 5.

## 4. Discussion

Coronary heart disease may be associated with cerebral small vessel lesions, which may contribute to ischemic changes and neuronal apoptosis, thereby potentially affecting cognitive function. Cognitive impairment and lower educational attainment may limit patients’ ability to understand and appropriately interpret supportive information from others. In addition, older age and lower educational level may restrict the postoperative social participation of older adults, which may in turn influence treatment adherence and their willingness to seek medical information 18. The present study showed that both perceived social support and quality of life were lower than normative levels [23,24], and the score of the confrontation dimension of medical coping (18.40 ± 3.58) was lower than that reported in previous studies [11]. We interpret these findings as reflecting the relatively vulnerable psychosocial and clinical status of the study population. Specifically, a large proportion of participants had low educational attainment, multiple chronic conditions, impaired cardiac function, and moderate to severe coronary artery disease, which may collectively weaken their coping capacity and psychosocial adaptation. The total health literacy score (76.69 ± 19.12) was comparable to that reported by Brørs [25] (76.3 ± 15.6), indicating a generally low level of health literacy. These findings suggest that older adults after PCI may still face challenges in acquiring and applying health-related knowledge, which may not be conducive to optimal recovery and improvement in quality of life.

It is important to clarify that hierarchical regression analysis and structural equation modeling were used for different analytical purposes in this study. The hierarchical regression model was primarily applied to assess multicollinearity and overall model stability (VIF < 5, significant F-test, and acceptable R^2^ values), rather than to evaluate mediation pathways [26]. In contrast, structural equation modeling was used to test the hypothesized theoretical framework, in which health literacy functions as a mediating variable rather than an independent predictor of quality of life. Therefore, the apparent differences in statistical significance between regression and SEM results reflect differences in analytical objectives rather than inconsistencies in the data.

In this study, the quality of life in older adults with coronary heart disease was assessed across multiple dimensions, including physical condition, disease status, medical condition, diet and sleep, psychological state, and interpersonal relationships. Cardiac function status and participation in cardiac rehabilitation were also considered important components. The main objective symptoms included angina pectoris, palpitations, and dyspnea, while psychosocial assessment mainly involved negative emotions, cognitive function, and social relationships. Perceived social support reflects patients’ subjective perception of external support and is closely related to psychosocial adaptation in older adults with chronic diseases. The results showed that perceived social support was positively associated with quality of life (r = 0.401, *p* < 0.01), suggesting that adults with higher levels of perceived support tend to report better overall health status and psychosocial well-being. This finding is consistent with previous studies [27,28]. We interpret this association as reflecting a buffering role of social support in reducing psychological stress and enhancing coping resources, which may be particularly relevant in older adults after PCI and may contribute to better psychological adaptation and quality of life [29].Based on these findings, improving patients’ perceived social support may be beneficial for postoperative recovery. Evidence has shown that cardiac rehabilitation programs may improve quality of life and psychosocial outcomes in patients with coronary heart disease [30]. For patients with limited mobility, hybrid rehabilitation models, including home-based cardiac rehabilitation and mobile health–supported interventions, may offer additional benefits by improving accessibility and enhancing self-management ability [31,32]. Therefore, healthcare professionals may consider integrating structured cardiac rehabilitation programs with psychological support interventions to support comprehensive postoperative recovery in older adults after PCI.

The mediating effect of the confrontation dimension of medical coping in the relationship between perceived social support and quality of life was 0.109, accounting for 17.38% of the total effect. Health literacy also demonstrated a partial mediating effect, with an effect size of 0.511, accounting for 81.50% of the total effect. These findings suggest that higher levels of perceived social support are associated with better quality of life, and that both coping strategies and health literacy may serve as important pathways linking psychosocial resources to health outcomes in older adults after PCI. As an important stress-coping resource, perceived social support may provide psychological energy and facilitate adaptation to disease-related stress, thereby contributing to improved quality of life [33]. Higher levels of perceived social support may help alleviate negative emotions and encourage patients to adopt more positive coping strategies [34], promoting a more adaptive attitude toward disease management [35]. In addition, health literacy appears to be a key pathway in this process, accounting for a substantial proportion of the total effect. It reflects patients’ ability to access, understand, and apply health-related information, and higher perceived social support may enhance these abilities by facilitating information exchange and the utilization of available support resources [14,36]. Therefore, strengthening perceived social support may be beneficial for promoting postoperative recovery and supporting cardiac rehabilitation in older adults with coronary heart disease.

Mediation analysis indicated that the confrontation dimension of medical coping and health literacy jointly formed a significant serial mediation pathway between perceived social support and quality of life. The indirect effect of the chain mediation was negative (β = −0.065), whereas the direct effect remained positive (β = 0.071), suggesting a suppression effect [37,38]. This pattern reflects a statistical phenomenon in which the inclusion of mediators alters the magnitude and direction of the total relationship due to the coexistence of opposing indirect pathways. Specifically, the suppression effect indicates that the total influence of perceived social support on quality of life is composed of both positive direct effects and negative indirect components through sequential mediation. Therefore, the observed suppression should be interpreted as a structural feature of the model rather than a causal contradiction. These findings suggest that the relationship among psychosocial resources, coping processes, and health literacy is complex and may not operate in a strictly additive manner. Further studies are needed to confirm the stability of this pathway in different populations.

This study has several limitations. First, only the confrontation dimension of the Medical Coping Modes Questionnaire was included based on a theoretical focus on problem-focused coping within the Stress–Appraisal–Coping framework. The exclusion of avoidance and acceptance–resignation dimensions may limit the comprehensiveness of coping assessment. Second, the cross-sectional design limits causal inference. Future multicenter longitudinal studies are needed to further validate these findings.

## 5. Conclusions

Perceived social support and quality of life among older adults with coronary heart disease undergoing PCI remain suboptimal. This study showed that perceived social support directly influences quality of life and also exerts indirect effects through the confrontation dimension of medical coping and health literacy. These findings highlight the need for healthcare professionals to strengthen patients’ perceived social support, promote positive coping during postoperative rehabilitation, and enhance health literacy to improve quality of life.

## Figures and Tables

**Table 1:** Patient Demographics and Quality of Life Scores (*n* = 353).

Project	Examples [n (%)]	Quality of Life Score (Score, $\bar{x}$ ± s)	*t*/*F*	*p*
Age (years)	-	-	23.708	<0.001
65~74	244 (69.1)	86.59 ± 18.97	-	-
75~84	98 (27.80)	72.95 ± 15.85	-	-
≥85	11 (3.10)	67.45 ± 15.56	-	-
Gender	-	-	4.493	<0.001
Male	245(69.41)	85.17 ± 18.01		
Female	108 (30.59)	75.47 ± 20.15	-	-
Educational Attainment	-	-	7.303	0.001
Elementary School or Below	214 (60.70)	79.15 ± 19.60		
Junior High School	93 (26.30)	86.19 ± 16.51	-	-
High School or Above	46 (13.00)	88.33 ± 19.77	-	-
Monthly Household Income (RMB)	-	-	9.988	<0.001
<3000	73 (20.70)	73.68 ± 18.14	-	-
3000~6000	204 (57.80)	83.77 ± 19.27	-	-
>6000	76 (21.50)	86.18 ± 17.73	-	-
Number of other chronic conditions	-	-	1.240	0.291
None	52 (14.70)	79.90 ± 17.82	-	-
1 condition	184 (52.10)	83.72 ± 21.05	-	-
≥2 conditions	117 (33.20)	80.85 ± 16.48	-	-
NYHA Functional Class	-	-	40.571	<0.001
Class I	191 (54.20)	89.57 ± 17.46	-	-
Class II	141 (39.90)	74.83 ± 17.39	-	-
Class III	21 (5.90)	64.76 ± 15.81	-	-
Severity of Coronary Artery Disease	-	-	7.791	<0.001
Mild Disease	57 (16.10)	91.21 ± 13.91	-	-
Moderate Disease	249 (70.50)	80.56 ± 19.40	-	-
Severe Disease	47 (13.40)	79.98 ± 20.82	-	-

Note: “-” indicates that the corresponding value is not applicable or not available.

**Table 2:** Patients’ Perceived Social Support, Medical Coping Dimensions, Health Literacy, and Quality of Life Scores (*n* = 353, $\bar{x}$ ± s, scores).

Project	Total Score	Entry	Entry Average Score
Perceived Social Support	54.50 ± 7.27	12	4.54 ± 0.61
Medical Coping Dimensions	18.40 ± 3.58	8	2.30 ± 0.45
Health Literacy	76.69 ± 19.12	27	2.84 ± 0.71
Quality of Life	82.20 ± 19.19	24	3.43 ± 0.80
Physical Condition	27.67 ± 11.59	2	13.83 ± 5.80
Disease Status	18.05 ± 3.36	6	3.01 ± 0.56
Medical Status	4.60 ± 1.34	2	2.30 ± 0.67
General Daily Life	9.34 ± 3.02	5	1.87 ± 0.60
Social and Psychologica	18.48 ± 3.84	7	2.64 ± 0.55
Work Capacity	4.11 ± 2.35	2	2.05 ± 1.18

**Table 3:** Pearson Correlation Analysis among Perceived Social Support, Confrontation Coping, Health Literacy, and Quality of Life (*n* = 353).

Variable	1	2	3	4
1. Perceived Social Support	1.000	-	-	-
2. Confrontation Coping	0.597 **	1.000	-	-
3. Health Literacy	0.399 **	0.521 **	1.000	-
4. Quality of Life	0.401 **	0.298 **	0.277 **	1.000

Note: 1 = Perceived Social Support; 2 = Confrontation Coping; 3 = Health Literacy; 4 = Quality of Life; Pearson Correlation Coefficient (** *p* < 0.01).

**Table 4:** Hierarchical Multiple Linear Regression Analysis of Factors Associated with Quality of Life (*n* = 353).

Independent Variable	Confrontation (Model 1)			Health Literacy (Model 2)			Quality of Life (Model 3)		
	β	*p*	VIF	β	*p*	VIF	β	*p*	VIF
Age	−0.107	0.039	1.236	0.051	0.249	1.251	−0.121	0.014	1.256
Sex	−0.048	0.345	1.212	−0.014	0.751	1.215	−0.053	0.266	1.215
Educational Attainment	0.205	<0.001	1.154	0.036	0.411	1.210	−0.002	0.969	1.213
NYHA Functional Class	−0.084	0.098	1.175	−0.056	0.195	1.185	−0.315	0.000	1.191
Severity	−0.033	0.487	1.060	0.056	0.174	1.061	−0.119	0.008	1.067
Perceived Social Support	0.307	<0.001	1.122	0.459	<0.001	1.248	0.313	<0.001	1.637
Confrontation	-	-	-	0.329	<0.001	1.332	0.069	0.199	1.532
Health Literacy	-	-	-	-	-	-	−0.024	0.687	1.850
F	19.117 ^①^			41.904 ^①^			23.021 ^①^		
R^2^	0.249			0.460			0.349		
Adjusted R^2^	0.236			0.449			0.334		

Note: ① indicates *p* < 0.001. Note: “-” indicates that the corresponding value is not applicable or not available.

**Table 5:** The Mediating Role of Health Literacy and Medical Coping Dimensions in the Relationship Between Perceived Social Support and Quality of Life.

Effect Relationship	β	SE	95%CI	*p*	Effect Proportion (%)
Total Effect	0.627	0.093	0.455~0.817	<0.001	100
Direct Effect	0.071	0.048	0.012~0.207	0.014	11.32
Total Indirect Effect	0.556	0.129	0.286~0.796	<0.001	88.68
Perceived Social Support → Confrontation → Quality of Life	0.109	0.031	0.052~0.174	<0.001	17.38
Perceived Social Support → Health Literacy → Quality of Life	0.511	0.173	0.191~0.873	0.003	81.50
Perceived Social Support → Confrontation → Health Literacy → Quality of Life	−0.065	0.053	−0.236~−0.015	0.002	91.55 ^①^

Note: ① denotes | indirect effect/direct effect |; “→” indicates the directional mediation pathways among variables in the model.

## Data Availability

The data presented in this study are available from the corresponding author upon reasonable request. The data are not publicly available due to privacy and ethical restrictions.

## Public Involvement Statement

No public involvement in any aspect of this research.

## Guidelines and Standards Statement

This manuscript was drafted in accordance with the STROBE Statement (Appendix A) for cross-sectional research.

## Appendix A

**Table A1:** STROBE Statement—Checklist of items that should be included in reports of cross-sectional studies.

	Item No	Recommendation	
**Title and abstract**	1	(*a*) Indicate the study’s design with a commonly used term in the title or the abstract	We have clarified in the title or the abstract section.
		(*b*) Provide in the abstract an informative and balanced summary of what was done and what was found	We have clarified in the abstract section.
**Introduction**			
Background/rationale	2	Explain the scientific background and rationale for the investigation being reported	Coronary heart disease (CHD) is the cardiac condition characterized by myocardial ischemia and hypoxia resulting from lumen narrowing, spasm, or obstruction caused by coronary atherosclerosis. With the acceleration of societal aging, individuals aged 65 and older now constitute 13.50% of China’s total population, and the prevalence of CHD within this cohort has reached as high as 20%. Percutaneous coronary intervention (PCI) serves as a key approach for managing coronary heart disease. Although it offers advantages such as minimal trauma and rapid recovery, patients face a sudden death risk 4 to 6 times higher than the general population post-procedure. Further-more, quality of life serves as a crucial indicator for assessing patient health status and plays a significant predictive role in treatment outcomes and disease prognosis. Additionally, quality of life is closely associated with patients’ ability to perceive social sup-port, coping strategies for illness, and health literacy. Perceived social support includes the emotional feelings and satisfaction of being valued, supported, and under-stood by other community members. It serves as a key factor in promoting psychosocial adaptation and is a crucial indicator of patients’ cognitive abilities. However, CHD pa-tients face a high risk of cognitive impairment, reaching up to 45%. Cognitive dysfunc-tion can weaken postoperative elderly patients’ comprehension and perception abili-ties. Resilience against neurodegenerative diseases and psychosocial motivators are crucial for maintaining brain cognitive function, enabling patients to actively engage in post-surgical cardiac rehabilitation. Medical coping strategies are characterized by cognitive adjustment and environmental adaptation. Their multidimensional approach enhances patients’ perception and utilization of social support, thereby improving health literacy levels. Health literacy is the capacity of a person to access, comprehend, and evaluate essential health information in order to make well-informed choices. Although associations exist between perceived social support, medical coping strategies, and quality of life, the pathway relationships and underlying mechanisms among these factors in elderly coronary heart disease patients undergoing PCI remain unexplored.
Objectives	3	State specific objectives, including any prespecified hypotheses	This study employs the Stress-Appraisal-Coping model to explore the underlying mechanisms linking perceived social support, medical coping strategies, health literacy, and quality of life among elderly patients undergoing PCI for coronary heart disease. The findings aim to provide evidence-based guidance for optimizing clinical care decisions and health management pathways.
**Methods**			
Study design	4	Present key elements of study design early in the paper	aged; coronary heart disease; percutaneous coronary intervention; perceived social support; mediating effect; cross-sectional study
Setting	5	Describe the setting, locations, and relevant dates, including periods of recruitment, exposure, follow-up, and data collection	A data collection team was established, consisting of one head nurse from the Department of Cardiology, one nurse in charge, and two postgraduate students. After obtaining informed consent, the questionnaires were administered one day before dis-charge using a one-to-one approach. The patients themselves filled out the questionnaires. In cases where patients faced challenges with writing, reading, or understand-ing, a trained investigator read each question aloud exactly as written and documented the responses based on the patients’ replies. The time needed for each participant to complete the questionnaire was roughly 10 to 30 min. Questionnaires were distributed and collected in person. Two researchers independently carried out data entry and conducted cross-checks to guarantee the accuracy, completeness, and validity of the information.
Participants	6	(*a*) Give the eligibility criteria, and the sources and methods of selection of participants	Older adults with coronary heart disease who were undergoing their initial PCI procedure in the Cardiovascular Department of a tertiary general hospital located in Anhui Province were selected through convenience sampling from September 2024 to April 2025. Inclusion criteria: (1) Age ≥ 65 years old; (2) First-time PCI procedure; (3) Informed consent and voluntary participation. Exclusion criteria: (1) New York Heart Association functional level IV; (2) Concurrent major organ diseases (e.g., cardiac, cerebral); (3) Inability to comprehend written text or communication barriers.
Variables	7	Clearly define all outcomes, exposures, predictors, potential confounders, and effect modifiers. Give diagnostic criteria, if applicable	We have clarified in the survey tool section (line 87~123).
Data sources/measurement	8 *	For each variable of interest, give sources of data and details of methods of assessment (measurement). Describe comparability of assessment methods if there is more than one group	We have clarified in the survey tool section.
Bias	9	Describe any efforts to address potential sources of bias	The investigation of common method bias was conducted through Harman’s single-factor test.
Study size	10	Explain how the study size was arrived at	According to the requirements for analyzing statistical variables, the sample size for a cross-sectional survey needs to be a minimum of 5 to 10 times greater than the number of variables involved. Taking the general information questionnaire (2 dimensions), the Perceived Social Support Scale (3 dimensions), the medical coping style facing dimension (1 dimension), the health literacy of percutaneous coronary intervention (3 dimensions), and the Quality of Life Assessment Questionnaire for Chinese Cardiovascular Patients (6 dimensions) as variables, a total of 15 variables were involved. And taking into account an 80% questionnaire validity rate requirement and based on a calculation that uses 10 times the number of variables, the minimum necessary sample size was determined to be 188 cases. Ultimately, 353 valid questionnaires were collected, meeting the structural equation modelling requirement of no fewer than 200 cases.
Quantitative variables	11	Explain how quantitative variables were handled in the analyses. If applicable, describe which groupings were chosen and why	Table 1.
Statistical methods	12	(*a*) Describe all statistical methods, including those used to control for confounding	All statistical evaluations were carried out with SPSS version 25.0.Continuous variables that adhered to a normal distribution were represented as mean ± standard. Group comparisons were executed using either the independent-samples t test or one-way analysis of variance, depending on the circumstances. Categorical variables were characterized by frequencies and their corresponding proportions. Pearson’s correlation analysis was utilized to investigate the relationships between perceived social support, confrontation coping, health literacy, and quality of life. To further explore these associations, structural equation modelling was conducted using AMOS version 26.0. In this analysis, the bootstrap resampling method was implemented to assess the mediating effects present among the variables studied. A mediating effect was deemed statistically significant if the 95% confidence interval did not encompass zero. The level of significance was established at α = 0.05, with a two-sided approach to ensure comprehensive results
		(*b*) Describe any methods used to examine subgroups and interactions	Group comparisons were executed using either the independent-samples t test or one-way analysis of variance, depending on the circumstances.
		(*c*) Explain how missing data were addressed	Questionnaires were distributed and collected in person. Two researchers independently carried out data entry and conducted cross-checks to guarantee the accuracy, completeness, and validity of the information.
		(*d*) If applicable, describe analytical methods taking account of sampling strategy	Elderly patients with coronary heart disease who were undergoing their initial PCI procedure in the Cardiovascular Department of a tertiary general hospital located in Anhui Province were selected through convenience sampling from September 2024 to April 2025. Inclusion criteria: (1) Age ≥ 65 years old; (2) First-time PCI procedure; (3) Informed consent and voluntary participation.
		(*e*) Describe any sensitivity analyses	The investigation of common method bias was conducted through Harman’s single-factor test. The exploratory factor analysis identified 14 factors that had eigenvalues exceeding 1. The initial unrotated factor explained 28.77% of the variance, which is be-low the conventional threshold of 40.00%. As a result, this study found no significant evidence of common method bias.
**Results**			
Participants	13 *	(*a*) Report numbers of individuals at each stage of study—eg numbers potentially eligible, examined for eligibility, confirmed eligible, included in the study, completing follow-up, and analysed	This research involved 353 senior patients suffering from coronary artery disease who received PCI, with ages between 65 and 91 years and a mean age of 71.91 ± 6.15 years. The univariate analysis indicated that factors such as age, gender, education level, monthly household income, heart function classification, and the severity of coronary artery disease significantly influenced patients’ quality of life, showing statistically meaningful differences (*p* < 0.05), as demonstrated in Table 1.
		(*b*) Give reasons for non-participation at each stage	NA
		(*c*) Consider use of a flow diagram	NA
Descriptive data	14 *	(*a*) Give characteristics of study participants (eg demographic, clinical, social) and information on exposures and potential confounders	Table 1 and Table 2.
		(*b*) Indicate number of participants with missing data for each variable of interest	A total of 355 questionnaires were distributed in this study, and 353 valid questionnaires were retrieved, with an effective recovery rate of 99.40%.
Outcome data	15 *	Report numbers of outcome events or summary measures	Figure 1 and Table 4.
Main results	16	(*a*) Give unadjusted estimates and, if applicable, confounder-adjusted estimates and their precision (eg, 95% confidence interval). Make clear which confounders were adjusted for and why they were included	NA
		(*b*) Report category boundaries when continuous variables were categorized	Table 1.
		(*c*) If relevant, consider translating estimates of relative risk into absolute risk for a meaningful time period	NA
Other analyses	17	Report other analyses done—eg analyses of subgroups and interactions, and sensitivity analyses	1.The exploratory factor analysis identified 14 factors that had eigenvalues exceeding 1. The initial unrotated factor explained 28.77% of the variance, which is below the conventional threshold of 40.00%. As a result, this study found no significant evidence of common method bias. 2.The univariate analysis indicated that factors such as age, gender, education level, monthly household income, heart function classification, and the severity of coronary artery disease significantly influenced patients’ quality of life, showing statistically meaningful differences (*p* < 0.05). 3.The overall quality of life measurement for older patients diagnosed with coronary heart disease who received PCI was 82.20 ± 19.19 points. The overall score for perceived social support was 54.50 ± 7.27 points, while the total score for the confrontation dimension of the Medical Coping Modes Questionnaire was 18.40 ± 3.58 points. Further-more, the health literacy score was 76.69 ± 19.12 points. 4.Pearson correlation revealed a positive association between quality of life and perceived social support, the confrontation aspect of medical coping, and health literacy, with correlation coefficients of r = 0.401, r = 0.298, and r = 0.277, respectively (all *p* < 0.01). Furthermore, both health literacy and the confrontation aspect exhibited a positive correlation with perceived social support, with correlation values of r = 0.399 and r = 0.597, respectively (both *p* < 0.01). Additionally, a positive correlation was observed between the confrontation aspect and health literacy (r = 0.521, *p* < 0.01). 5.Multivariate Linear Hierarchical Regression Analysis of Variable Relationships in Chain-of-Agency Models: the variance inflation factors (VIF < 5) met the required criteria, indicating that there was no multicollinearity among the variables in each model. 6.Following the adjustments made to the model, the indices indicating goodness-of-fit were as follows: χ^2^/df = 3.127, NFI = 0.959 (>0.900), IFI = 0.972 (>0.900), TLI = 0.948 (>0.900), and RMSEA = 0.078 (<0.080). Each of these indices satisfied the suggested thresholds, signifying a strong fit for the model. 7.In this study, the 95% CI for all three pathways between perceived social support and quality of life excluded zero, indicating significant mediating effects. The indirect effect accounted for 17.38% (0.109/0.627) of the total effect in the pathway perceived social support → confrontation dimension → quality of life, and 81.50% (0.511/0.627) in the pathway perceived social support → health literacy → quality of life. When the confrontation dimension and health literacy were included as sequential mediators, the indirect and direct effects had opposite signs, indicating a suppression effect. The ratio of the suppression effect, determined by taking the absolute value of the indirect effect and dividing it by the direct effect, was calculated to be 91.55% (|−0.065/0.071|).
**Discussion**			
Key results	18	Summarise key results with reference to study objectives	Perceived social support and quality of life among elderly patients with coronary heart disease undergoing PCI remain suboptimal. This study showed that perceived social support directly influences quality of life and also exerts indirect effects through the confrontation dimension of medical coping and health literacy. These findings highlight the need for healthcare professionals to strengthen patients’ perceived social support, promote positive coping during postoperative rehabilitation, and enhance health literacy to improve quality of life.
Limitations	19	Discuss limitations of the study, taking into account sources of potential bias or imprecision. Discuss both direction and magnitude of any potential bias	And the study has limitations. The use of convenience sampling may have introduced information bias. Future mu-lticenter studies with larger samples are needed to further examine the dynamic relationships among perceived social support, coping styles, health literacy, and quality of life, in order to optimize comprehensive interventions, prevent postoperative complications, and promote rehabilitation outcomes.
Interpretation	20	Give a cautious overall interpretation of results considering objectives, limitations, multiplicity of analyses, results from similar studies, and other relevant evidence	Based on the pressure-evaluation-coping model, this study explores the internal mechanism and action path among perceived social support, the confrontation dimension of medical coping style, health literacy and quality of life in elderly patients with coronary heart disease after PCI. In this research, PCI is regarded as the stressor for elderly patients with coronary heart disease, perceived social support and health literacy are the key factors influencing patients’ cognitive evaluation, and the quality of life level is the ultimate outcome of patients’ stress and coping process. Thus, a path model is constructed with perceived social support as the independent variable, the confrontation dimension of medical coping style and health literacy as the mediating variables, and quality of life as the dependent variable, providing a theoretical basis for improving the quality of life of postoperative patients.
Generalisability	21	Discuss the generalisability (external validity) of the study results	Medical coping style and health literacy play a chain mediating role between perceived social support and quality of life. That is, perceived social support can directly and positively predict the quality of life of elderly patients with coronary heart disease after PCI surgery, and it can also indirectly affect the quality of life of postoperative patients through the partial mediating effect of medical coping style or health literacy or the chain mediating effect.
**Other information**			
Funding	22	Give the source of funding and the role of the funders for the present study and, if applicable, for the original study on which the present article is based	Project of Anhui Provincial Health Commission (2024Aa10044); Young Talent Project of Anhui Medical University (Hlqm12025109).

* Give information separately for cases and controls in case-control studies and, if applicable, for exposed and unexposed groups in cohort and cross-sectional studies.

## References

[1] Wu H., Wang Y., Zhang H., Yin X., Wang L., Wang L., Wu J. (2024). An investigation into the health status of the elderly population in China and the obstacles to achieving healthy aging. Sci. Rep..

[2] Martin S.S., Aday A.W., Almarzooq Z.I., Anderson C.A., Arora P., Avery C.L., Baker-Smith C.M., Barone Gibbs B., Beaton A.Z., Boehme A.K. (2024). 2024 heart disease and stroke statistics: A report of us and global data from the american heart association. Circulation.

[3] Chai Z., Fan Y., Gong X., Zhang Y., Hu Y., Li X., Fan Z., Han Y. (2025). Adherence to phase I cardiac rehabilitation in post-PCI patients: A latent class analysis. Front. Cardiovasc. Med..

[4] Van Nguyen H., Khuong L.Q., Nguyen A.T., Nguyen A.L.T., Nguyen C.T., Nguyen H.T.T., Tran T.T.H., Dao A.T.M., Gilmour S., Van Hoang M. (2021). Changes in, and predictors of, quality of life among patients with unstable angina after percutaneous coronary intervention. J. Eval. Clin. Pract..

[5] Frøjd L.A., Munkhaugen J., Papageorgiou C., Sverre E., Moum T., Dammen T. (2023). Predictors of health-related quality of life in outpatients with coronary heart disease. Front. Psychol..

[6] Fan Y., Shen B.J., Ho M.R. (2024). Loneliness, perceived social support, and their changes predict medical adherence over 12 months among patients with coronary heart disease. Br. J. Health Psychol..

[7] Shu P., Xuan L., Jiang X. (2024). Discharge readiness and associated factors among patients with coronary heart disease after stent implantation: A cross-sectional single center study. Patient Prefer. Adherence.

[8] Kähkönen O., Paukkonen L., Vähänikkilä H., Oikarinen A. (2024). Perceived social support among percutaneous coronary intervention patients over a long-term follow-up period. Nurs. Open.

[9] Wakim M.L., Al Haddad C., Haddad C., Mouawad M., Hachem D. (2025). The relationship between perceived social support and quality of life among hospitalized patients with schizophrenia. Sci. Rep..

[10] Quiroz Y.T., Aguillón D., Arboleda-Velasquez J., Bocanegra Y., Cardona-Gómez G.P., Corrada M.M., Diez I., Garcia-Cifuentes E., Kosik K., Martinez L. (2025). Driving research on successful aging and neuroprotection in Latin America: Insights from the inaugural symposium on brain resilience and healthy longevity. Alzheimers Dement..

[11] Liang L.X., Liu Y., Shi Y.J., Jiang T.T., Zhang H.R., Liu B.H., Xu P.Z., Shi T.Y. (2022). Family care and subjective well-being of coronary heart disease patients after percutaneous coronary intervention: Mediating effects of coping strategies. Int. J. Nurs. Sci..

[12] Moon S.H., Jeong H.W., Jung U.S. (2024). Exploring the impact of the mentoring new nurses for transition and empowerment program led by clinical nurse educators in South Korea: A mixed-methods study. Nurse Educ. Today.

[13] Meraz R., Osteen K., McGee J.S., Noblitt P., Viejo H. (2023). Applying stress and coping theory to understand diuretic adherence experiences in persons with heart failure. West. J. Nurs. Res..

[14] Baker D.W. (2006). The meaning and the measure of health literacy. J. Gen. Intern. Med..

[15] Liu W.Q., Yang W.L., Qian S.Y. (2023). The mediating effect of self-efficacy on health literacy and social support in young and middle-aged patients with coronary heart disease after PCI. Vasc. Health Risk Manag..

[16] Montazeri A.S., Azizi Z., Ranjbar L., Rezaei S., Zare A. (2026). Associations between perceived social support, self-efficacy, and health-promoting behaviors in hospitalized heart failure patients: A cross-sectional study. BMC Cardiovasc. Disord..

[17] Gensini G.G. (1983). A more meaningful scoring system for determining the severity of coronary heart disease. Am. J. Cardiol..

[18] Zimet G.D., Powell S.S., Farley G.K., Werkman S., Berkoff K.A. (1990). Psychometric characteristics of the multidimensional scale of perceived social support. J. Personal. Assess..

[19] Huang L., Jiang Q.J., Ren W.H. (1996). Correlation between coping styles, social support and psychosomatic symptoms in cancer patients. Chin. Ment. Health.

[20] Feifel H., Strack S., Nagy V.T. (1987). Coping strategies and associated features of medically ill patients. Psychosom. Med..

[21] Shen X.H., Jiang Q.J. (2000). Test report on the Chinese version of the Medical Coping Modes Questionnaire in 701 patients. Chin. J. Behav. Med. Sci..

[22] Yue M., Zhang L.J., Lu Y.Y. (2023). Development and validation of a health literacy scale for patients undergoing percutaneous coronary intervention. J. Nurs. Sci..

[23] Liu J.S., Ma C.M., Tu L.Z., Wang Y., Zheng B.R., Wang F.J., Hong H.S., Guo L., Yin Z.F., Li P.H. (2012). Development of the Chinese Cardiovascular Patients’ Quality of Life Questionnaire and establishment of normative data. Chin. J. Cardiovasc. Rehabil. Med..

[24] Ifroh R.H., Gai X., Rabiautsani M.A., Han X. (2024). The social support, healthy lifestyle, subjective well-being, and health-related quality of life among university students. J. Educ. Health Promot..

[25] Brørs G., Dalen H., Allore H., Deaton C., Fridlund B., Norman C.D., Palm P., Wentzel-Larsen T., Norekvål T.M. (2023). The association of electronic health literacy with behavioural and psychological coronary artery disease risk factors in patients after percutaneous coronary intervention: A 12-month follow-up study. Eur. Heart J. Digit. Health.

[26] Hair J.F., Black W.C., Babin B.J., Anderson R.E. (2019). Multivariate Data Analysis.

[27] Zhao J., Hu D., Du H., Wang H., Tu X., Wang A. (2025). Social support and technophobia in older patients with coronary heart disease: The mediating roles of eHealth literacy and healthcare technology self-efficacy. PLoS ONE.

[28] Ding Y., Wang X., Zhang F., Yan H., Liu Y., Zhang L. (2024). The relationship between perceived social support, coping style, and the quality of life and psychological state of lung cancer patients. BMC Psychol..

[29] Lok N., Buldukoglu K., Barcin E. (2020). Effects of the cognitive stimulation therapy based on Roy’s adaptation model on Alzheimer’s patients’ cognitive functions, coping-adaptation skills, and quality of life: A randomized controlled trial. Perspect. Psychiatr. Care.

[30] Moreira J., Bravo J., Aguiar P., Delgado B., Raimundo A., Boto P. (2024). Physical and mental components of quality of life after a cardiac rehabilitation intervention: A systematic review and meta-analysis. J. Clin. Med..

[31] Candjondjo P., Vaz E., Marques S., Moreira J. (2022). Stemi-cr: Solution for patients with acute myocardial infarction at home-based cardiac rehabilitation program. Procedia Comput. Sci..

[32] Miguel S.S., Frade A.I., Moreira J.M. (2024). The use of m-health to improve self-care in patients with heart failure. Eur. J. Cardiovasc. Nurs..

[33] Thapa A., Kang J., Chung M.L., Wu J.R., Biddle M.J., Cha G., Moser D.K. (2022). Self-care, perceived social support, and health-related quality of life in persons with heart failure. Nurs. Res..

[34] Guan X., Zhu Q., Qian H. (2025). Relationship between post-traumatic stress disorder and fear of progression in stroke patients: The mediating role of perceived social support and coping styles. Top. Stroke Rehabil..

[35] Wang L., Zhou B., Wang L. (2024). Effect of care bundles based on importance degree analysis on postoperative comorbid state, coping style and disease management ability of patients with coronary heart disease. Medicine.

[36] Jennings C.S., Astin F., Prescott E., Hansen T., Chris P.G., Bacquer D.D. (2023). Illness perceptions and health literacy are strongly associated with health-related quality of life, anxiety, and depression in patients with coronary heart disease: Results from the euroaspire v cross-sectional survey. Eur. J. Cardiovasc. Nurs..

[37] MacKinnon D.P., Krull J.L., Lockwood C.M. (2000). Equivalence of the mediation, confounding and suppression effect. Prev. Sci..

[38] Hayes A.F. (2018). Introduction to Mediation, Moderation, and Conditional Process Analysis: A Regression-Based Approach.

